# Integration of phytochemicals and phytotherapy into cancer precision medicine

**DOI:** 10.18632/oncotarget.17466

**Published:** 2017-04-27

**Authors:** Thomas Efferth, Mohamed E.M. Saeed, Elhaj Mirghani, Awadh Alim, Zahir Yassin, Elfatih Saeed, Hassan E. Khalid, Salah Daak

**Affiliations:** ^1^ Department of Pharmaceutical Biology, Institute of Pharmacy and Biochemistry, Johannes Gutenberg University, Mainz, Germany; ^2^ Salah Wanasi Foundation for Cancer Research and Control, Khartoum, Sudan; ^3^ Faculty of Medicine, International University of Africa, Khartoum, Sudan; ^4^ Tayba Cancer Centre, Khartoum, Sudan; ^5^ Federal Government of Sudan, Khartoum, Sudan; ^6^ Department of Pharmacognosy, University of Khartoum, Khartoum, Sudan

**Keywords:** drug resistance, network pharmacology, polypharmacology, targeted chemotherapy

## Abstract

Concepts of individualized therapy in the 1970s and 1980s attempted to develop predictive *in vitro* tests for individual drug responsiveness without reaching clinical routine. Precision medicine attempts to device novel individual cancer therapy strategies. Using bioinformatics, relevant knowledge is extracted from huge data amounts. However, tumor heterogeneity challenges chemotherapy due to genetically and phenotypically different cell subpopulations, which may lead to refractory tumors. Natural products always served as vital resources for cancer therapy (*e.g*., *Vinca* alkaloids, camptothecin, paclitaxel, etc.) and are also sources for novel drugs. Targeted drugs developed to specifically address tumor-related proteins represent the basis of precision medicine. Natural products from plants represent excellent resource for targeted therapies. Phytochemicals and herbal mixtures act multi-specifically, *i.e*. they attack multiple targets at the same time. Network pharmacology facilitates the identification of the complexity of pharmacogenomic networks and new signaling networks that are distorted in tumors. In the present review, we give a conceptual overview, how the problem of drug resistance may be approached by integrating phytochemicals and phytotherapy into academic western medicine. Modern technology platforms (*e.g*. “-omics” technologies, DNA/RNA sequencing, and network pharmacology) can be applied for diverse treatment modalities such as cytotoxic and targeted chemotherapy as well as phytochemicals and phytotherapy. Thereby, these technologies represent an integrative momentum to merge the best of two worlds: clinical oncology and traditional medicine. In conclusion, the integration of phytochemicals and phytotherapy into cancer precision medicine represents a valuable asset to chemically synthesized chemicals and therapeutic antibodies.

## INTRODUCTION

Resistance to anticancer drugs already appeared in the very early days of cancer chemotherapy more than half a century ago [1–6], and it still hampers successful treatment of patients nowadays [7]. Many established anticancer drugs kill proliferative cells, whether or not they are malignant. This approach causes only modest tumor specificity, and non-tumorous normal proliferative tissues are also affected. Thereby, the application of drug doses high enough to kill all tumor cells including the less sensitive tumor subpopulations cannot be applied without provoking severe side effects in cancer patients. As a consequence, sub-optimal drug doses may let few inherently resistant tumor cells unaffected, which subsequently grow out leading to the reappearance of tumors. These refractory tumors do not respond to cytostatic therapy anymore with fatal outcomes for patients.

Current chemotherapy protocols are based on the result of prospective, randomized, double-blind phase III studies, which results in similar clinical standard treatment guidelines. However, each tumor may behave in a different manner and the treatment success of individual patients still cannot be reliably predicted, although the statistical probability of treatment response for larger groups of patients can be estimated from the results of clinical trials. The reason is that even tumors of the same origin and histology may differ from patient to patient in their biological behaviour. Even more, cells of the same tumor may be different from each there, and there is a substantial heterogeneity which greatly influences the response of tumor cell subpopulations to chemotherapy. While some subpopulations respond well to treatment, others resist and give rise to treatment failure. The appearance of drug resistance is a major, still unresolved obstacle in cancer therapy even after many decades of enormous efforts in cancer research. It has therefore been attempted to understand the molecular mechanisms of drug resistance and to predict *a priori*, whether or not an individual tumor would respond to drug therapy and to adapt treatment according to the individual drug sensitivity profile of tumors [8]. Whereas sensitivity or resistance to targeted drugs (*e.g*., HER2- or estrogen-receptor-targeting small molecules or therapeutic antibodies) may be straightforward, the reliable prediction of treatment efficacy for the clinically long established cytotoxic drugs is much more complicated, since their cellular targets frequently less well-defined and these kinds of cytostatic drugs reveal broader modes of action against malignant and even normal proliferating cells.

**Figure 1:**
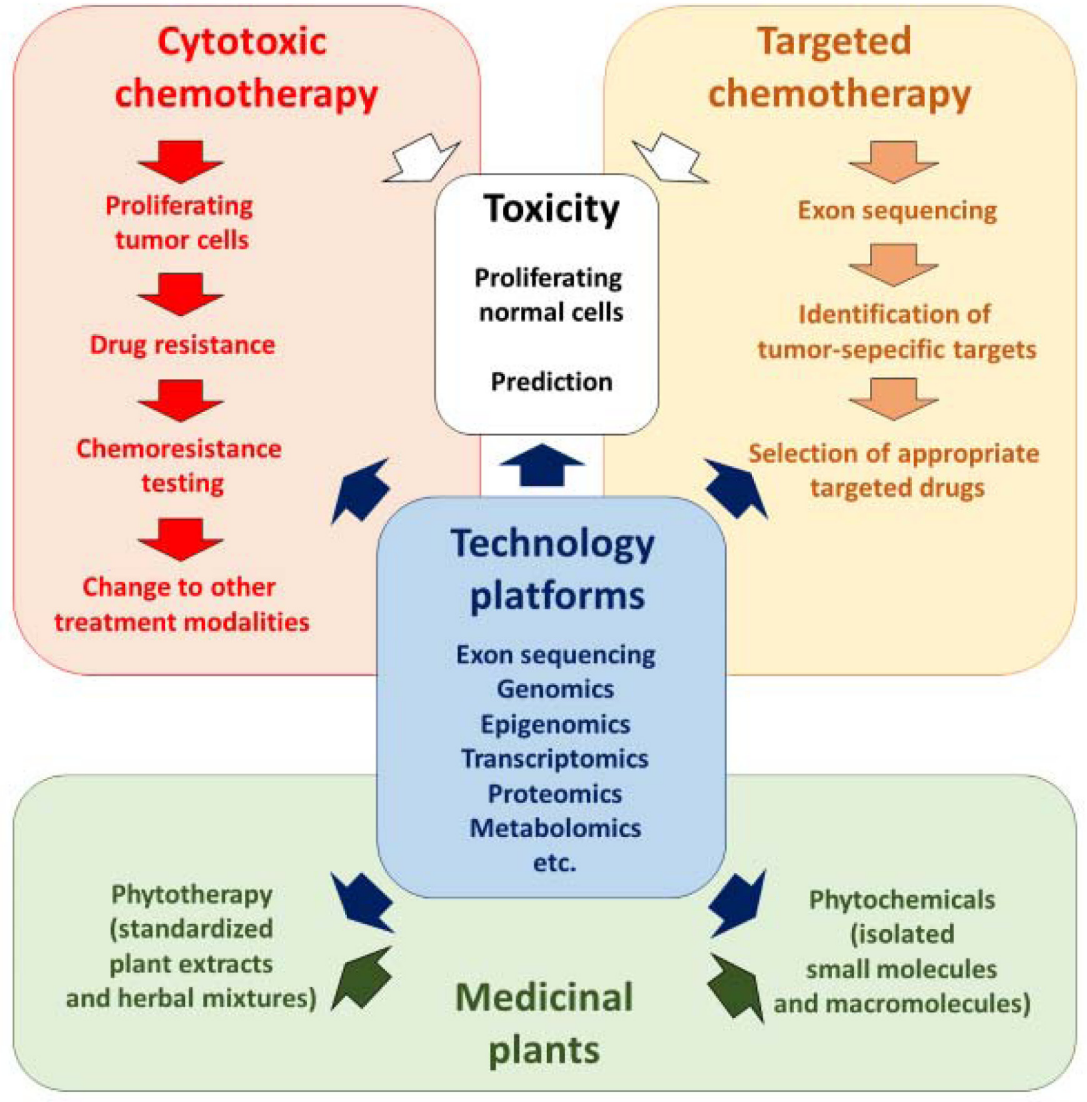
Integration of phytochemicals and phytotherapy into standard academic oncology

In the present review, we give a conceptual overview, how the problem of drug resistance may be approached by integrating phytochemicals and phytotherapy into academic western medicine. Modern technology platforms (*e.g*. “-omics” technologies, DNA/RNA sequencing, and network pharmacology) can be applied for diverse treatment modalities such as cytotoxic and targeted chemotherapy as well as phytochemicals and phytotherapy (Figure 1). Thereby, these technologies represent an integrative momentum to merge the best of two worlds: clinical oncology and traditional medicine.

## PREDICTION OF DRUG SENSITIVITY AND RESISTANCE

Diagnostic tests are desirable to predict sensitivity of resistance of each individual tumor to drug treatment. If a tumor is resistant, therapy may only provoke toxicity in healthy organs without eradication the cancer itself [6]. In case a tumor is resistant to certain drugs, the treatment regimen may be changed to other still effective drugs. Therefore, early concepts of individualized therapy in the 1970s and 1980s attempted to devise diagnostic *in vitro* tests to predict the drug responsiveness in individual patients [9, 10]. Although many different assays have been developed, none of them reached the status of routine clinical diagnostics [5, 11]. A meta-analysis of the literature published during the past four decades considering test results of more than 15, 000 tumor patients unambiguously demonstrated in the majority of studies that resistance was correctly predicted with accuracy between 80 and 100%, while drug sensitivity could only be predicted with an accuracy of 50-80% [10]. In the past decades, the main attention of oncologist was focused on the identification of sensitive drugs which would be able to treat otherwise resistant tumors. Therefore, the high reliability of these *in vitro* tests to predict resistance rather than sensitivity was not sufficiently appreciated. This was mainly due to the lack of alternative treatment options in case a tumor turned out to be resistant to the available standard drugs at that time. The physicians in the clinics were understandably reluctant to tell a patient “Your tumor is predicted as being resistant. Sorry, we cannot do anything for you”. Nowadays the situation has changed as more treatment strategies are available. If a tumor behaves resistant to one or several cytostatic anticancer drugs, other options may be chosen such as antibody therapy, adoptive immunotherapy, high-dose therapy, hematopoietic stem cell transplantation, supportive gene therapy protocols, hyperthermia, and also - as we will see below - phytotherapy. Therefore, the high reliability to predict resistance should be understood as a valuable chance to plan other individualized treatment options. Therefore, a rethinking of the “chemosensitivity” concept has been proposed to unravel and revive the full potential of a revival of predictive tests for personalized medicine.

The concept of predictive chemosensitivity and -resistance testing has not been documented in the literature for natural products and herbal remedies by far to the same extent as it did for standard cancer chemotherapy [12]. In traditional Chinese medicine (TCM), where individualized therapy is one of the most prominent and important features, the decision on the right combination of herbs for an individual cancer patient mainly relies on the particular TCM diagnostics rather than on predictive *in vitro* tests. Therefore, these kinds of assays are not very popular. In Western countries, predictive chemosensitivity assays are broadly offered by commercial sources for use in complementary and alternative medicine. Although it can well happen that they will frequently be applied, results are scarcely reported in the scientific literature.

## RELEVANCE OF “-OMICS” TECHNOLOGIES FOR PRECISION MEDICINE AND DRUG DISCOVERY

A crucial decision in individualized therapy is not only the choice of the right drug for the right patient, but also the right combination of drugs and the duration and dose of treatment. Because of the complexity of tumor genomes, this may be difficult to dissect with the classical methodology of clinical trials. As a direct consequence of the deciphering of the human genome at the verge from the second to the third millennium, techniques have been developed that allow to determine (1) genomic variations such as single nucleotide polymorphisms (SNPs) [13], copy number variations and other structural variations associated with disease progression and drug response (genomics), (2) epigenetic modifications such as DNA-methylation, histone acetylation or micro-RNA expression (epigenomics), (3) transcriptome-wide mRNA expression (transcriptomics), (4) proteome-wide protein and peptide expression (proteomics) in cells or tissues. These methods have frequently been termed “-omics” technologies. In recent years, the rapid technological advancements brought transcriptome-wide RNA-sequencing (“next generation sequencing”) into play. The results obtained with these sophisticated techniques are evaluated by methods of computational biology and bioinformatics to extract and model the relevant knowledge gained from a vast plethora of generated data [14, 15]. The potential of this new technological dimension of technology lies in its translation from the laboratory to practical routine for diagnosis and treatment and comprehensive approach to diagnosing tumors and tumor subtypes, to predict response to treatment and occurrence of unwanted side effects), Individualized treatment regimens may be planned based on a patient's (or tumor's) individual expression profiles to optimize the survival prognosis of cancer patients [16–18]. Several conditions determine the setup of patient-tailored therapies, *e.g*. (1) comprising meta-analyses on DNA sequencing results and related implications for drug development, and (2) the availability of individual targeted agents and biomarkers for therapy monitoring [19]. A multi-dimensional clinical genomics study of children and adolescent young adults with relapsed non-central nervous system solid cancers may be taken as a clue, how integrative genomic analyses and robust bioinformatics may serve to generate precision therapy protocols for the future [20].

The challenge of this new concept of precision medicine will be to delineate individual and efficient cancer therapy strategies, which are superior to traditional concepts of standardized tumor treatment. Enormous amounts of “-omics” data together with drug combination studies and biological network data analyses are required, since tumors consist of heterogeneous subpopulations with distinct biological features [21, 22]. A pilot project in this context represents the connectivity map, which aims to establish a connection between chemicals and gene expression profiles in different cancer cell lines for more than 1700 compounds [23]. This project is certainly only a starting point, but it illustrates the complex requirements to develop individualized treatment protocols.

Advancements in molecular diagnostics is only one side of the coin, and the development of novel drugs has to keep pace, which may be an even more difficult task to master. Drug development and marketing is a time- and cost-intensive process and the number of newly approved drugs declined for decades, mainly because of their failure in clinical phase 2 trials, despite the fact that time and expenditure on drug research and development (R&D) consistently increased during recent years [24, 25]. The hope was that the emerging high throughput technologies might supplement “-omics” technologies and next generation sequencing to generate novel drug candidates for the market.

In this context, a surprising concept emerged [26]. Existing drugs with a well-known safety and pharmacokinetic profiles that failed against certain diseases might serve as valuable drug candidates for other diseases affected by the same pathway. This phenomenon has been described as drug repositioning [27]. An intriguing example of the potential of drug repositioning is thalidomide, which has been banned as barbiturate for its teratogenic effects [28]. Later on, thalidomide has been identified as effective drug against severe erythema nodosum leprosum [29] and multiple myeloma [30]. Another less dramatic example is retinoic acid, which has been found to be active against acute promyelocytic leukemia [31].

## TUMOR HETEROGENEITY

Cancers of the same histological type do not only differ from patient to patient, but also consist of heterogeneous subpopulations of cells within one and the same tumor. Heterogeneity represents a considerable challenge to cancer chemotherapy, since it aggravates the effective eradication of all cells of genetically and phenotypically different subpopulations. Even few surviving tumor cells may lead to repopulation and refractory tumors. Single-cell sequencing allows novel insights into the diversified and complex molecular architecture of heterogeneous tumors. The isolation and sequencing of single tumor cells are technically very challenging and consists of three major steps: (1) single cell isolation (*e.g.* by laser-capture microdissection or fluorescence-activated cell sorting), (2) whole genome amplification (*e.g*. with the help of Phi29 DNA polymerase), and (3) transcriptome-wide next generation sequencing technologies. The problem of tumor heterogeneity especially applies to drug resistance. Single cell sequencing will facilitate the detection of even the smallest populations of drug-resistant cells. Thereby, single-cell sequencing and may form the basis for novel and improved treatment options to eradicate drug-resistant tumor cells with specific genetic alterations [32].

## THE CONTRIBUTION OF NATURAL PRODUCTS

Natural products always served as vital resources for cancer therapy. Well-known examples of the therapeutic potential of plant-derived natural products plants are the microtubule inhibiting *Vinca* alkaloids, the DNA topoisomerase I inhibitor camptothecin, the terpene paclitaxel, or the podophyllotoxin-derived lignans, etoposide and teniposide.

A survey of the National Cancer Institute showed that 69% of anticancer drugs approved between the 1980s and 2002 are either natural products or developed based on knowledge gained from natural products [33]. Intriguingly, about three-quarters of plant-derived drugs in clinical use nowadays have their roots in traditional phytomedicines.

Although medicinal herbs belonged to the standard repertoire to combat diseases since ages, they gradually lost relevance with increasing successes of synthetic pharmaceuticals in western countries during the 20^th^ century. Owing to the interest in bioactive natural products as chemical lead compounds for the generation of semi-synthetic derivatives with improved pharmacological features, medicinal plants as well as other natural resources (from marine or microbiological ecosystems) experience a thriving revival [34–36].

Secondary metabolites are synthesized by plants as a defense against competitors, herbivores and pathogens and as signal compounds that attract insects for reproduction. Repelling predators is crucial for plants since they do not have elaborated immunological defense mechanisms nor can they flew, because they are sessile. Secondary metabolites maintain crucial functions for survival and reproductive fitness of plants [37, 38]. In addition to toxicity, secondary plant metabolites exert pharmacological features, which make them valuable for treatment purposes. The separation of these beneficial from harmful effects is the goal of modern and pharmacognosy and pharmacology/toxicology [39, 40].

## THE CHALLENGE POSED BY PHYTOTHERAPY

The conferment of the Nobel Award for Physiology or Medicine 2015 was Youyou Tu and her life-time achievements on artemisinin from the Chinese medicinal plant, *Artemisia annua* L. for malaria therapy represents and appreciation for the entire scientific community working on phytotherapy and natural products [41]. Therefore, it did not come as a surprise that the Nobel Award to Youyou Tu was anticipated with much enthusiasm. Now, when the celebrations are over and the grey everyday work reality is returning back, it is the right time to address the question what is coming next and how to continue from here. In other words, which sustainable actions are needed to come to long-lasting and significant improvements in TCM and phytotherapy in general for the sake of patients?

Research and development on artemisinin as malaria medication followed the rules and strategies of classical pharmacological drug, but not those of the development of a typical phytotherapeutic drug. The process ended with a chemical substance rather than with a standardized herbal product. The same applies for many other drugs which became established parts of modern pharmacopoeias. A majority of modern drugs, which are derived in one or the other way from natural sources were only inspired by nature [33], but not phytotherapies in a strict sense.

As a matter of fact, traditional medicine is being applied million times on this globe, and therefore the general conditions are basically different from those of synthetic chemical drugs. This may be an advantage, but represents a disadvantage at the same time, because there is less burning economic pressure to fulfil the strict regulations of the drug-approval authorities. If a herbal preparation is not marketed as drug by a pharmaceutical company, it can be either sold over the counter as dietary supplement without fulfilling any quality control measures, or it can be used as therapeutic drug for individual compassionate uses. These practices demonstrate that herbal medicines are popular among patients, but they frequently do not provide sufficient evidence that they are safe and efficient.

As popular herbal medicines are among physicians, practitioners and patients all over Asia, as critical they are considered among the medical community in the West. Several reasons can be discussed to explain this contradiction. One problem is certainly that herbal medicine does not belong to the standard repertoire of knowledge that is taught in our medical schools to the students. This is a fatal situation to our point of view. Another critical issue is, however, that the safety and quality of herbal products has still not been proven to the same extent, as it is routinely done for synthetic drugs.

The medical use of plants by traditional healers is frequently accompanied by spiritual and magical rituals. While these practices are doubtless of interest to cultural anthropology, rationale phytotherapy should focus solely on seeking scientific evidence for the pharmacological activity of medicinal plants. On the other hand, the demystification and “secularization” of medicinal plants also applies to industrialized countries, where “green medicine” is sometimes associated with esoteric elements.

If we consider phytotherapy as a regular discipline of life science, which rules and methods should be applied to do so? Crucial elements of quality control include [42]

- ethnobotany: documentation of traditional knowledge;
- botanical verification of medicinal herbs (modern taxonomy, HPLC fingerprinting, DNA sequencing);
- standardization of herbal products and mixtures;
- avoidance of contaminations and adulterations;
- elucidation of molecular and cellular modes of action;
- placebo-controlled, double-blind and randomized clinical trials.

To guarantee the marketing of safe and efficacious herbal products, national and international authorities regulate the approval of these medicines, *e.g*. the Food and Drug Administration (FDA) in the United States and the European Medicines Agency (EMA), both of which enforce regulatory sets of specifications including the quality, purity standards, dosage, production, precautions, storage, and labeling of these medicines [43]. Monographs provide the specifications of each plant. These monographs are usually compiled in pharmacopoeias which are considered as official documents specifying the quality, purity standards, dosage, production, precautions, storage, and labeling of medicines.

A main goal of the utilization of these diverse genetic resources is to market herbal products (*i.e*. bioprospecting). Since the use of medicinal plants is mainly based on the traditional knowledge of indigenous communities, commercialization of herbal products by pharmaceutical industries should, follow fair rules of benefit-sharing with those, where the knowledge comes from. Biopiracy practices by multinational companies in the past should be banned [44]. The World Health Organization (WHO) and the United Nations Educational, Scientific and Cultural Organization (UNESCO) draw the international attention on the protection of indigenous knowledge. The Nagoya protocol contains rules how to protect traditional medical knowledge and to compensate indigenous people for knowledge that is already being patented or being used in an inappropriate manner in the past. The European Parliament accepted the protocol (EU, No. 511/2014) on April 16^th^ 2014. It entered into force on October 12^th^ 2014. Main principles are (1) informed consent of the country of origin of the resource and (2) mutually agreed terms between indigenous peoples and collaborative commercial partners.

Summing up, the future of phytotherapy lies without doubt in the production of high-quality products, which are able to compete with synthetic drugs regarding safety and efficacy. Phytotherapy must not be mixed up with quackery practices in alternative medicine. Rationale phytotherapy should fight for its image as effective medicine with good tolerability.

## TARGETING TUMOR-RELATED PROTEINS WITH NATURAL PRODUCTS

The problem with classical standard chemotherapy is that drugs do not sufficiently distinguish between normal and malignant growing cells. As long as cells are dividing, they are attacked by the drugs. A new concept is to seek for molecular differences between normal and cancerous cells and specifically attack cancer-related targets by drugs. Targeted drugs are designed to kill cancer cells by their binding to the target. Since this target is not present in normal cells, targeted cells are expected not to exert side effects on normal organs. Sophisticated techniques are used to identify targets for cancer therapy such as “-omics” technologies, cytogenetic methods, etc. Frequently, tumor cells bear amplified genes leading to protein overexpression are not present in normal cells. Also, chromosomal translocations may generate fusion genes coding for novel fusion proteins that are not existing in healthy cells. Some of these aberrantly expressed genes drive the development of cancer if they contain oncogenic sequences (*driver genes*). Other genetic aberrations occur in the course of tumor progression as a consequence of genomic instability of tumors. They do not have a major impact on the malignancy of cancer (*passenger genes*). Targeted drugs developed to specifically address proteins encoded by driver genes represent the basis for individualized or precision medicine. Instead of standardized therapy regimens for all patients suffering from the same tumor type, targeted drugs can be individually used depending on the specific expression of aberrant targets in each single patient. Two main categories of targeted drugs have been developed: (1) monoclonal antibodies that address cell-surface proteins. Examples are cetuximab and panitumumab against the epidermal growth factor receptor (EGFR), bevacizumab against vascular endothelial growth factor (VEGF) and rituximab against CD20. (2) Small chemical molecules that easily can enter tumor cells to attack intracellular targets (*e.g*. cancer-related kinases). Imatinib mesylate is a showcase example of historical relevance for the proof-of-principle of the entire concept of targeted therapy. This drug binds and inhibits the oncogenic BCR/ABL fusion protein. Other small molecules are erlotinib and gefitinib against EGFR, vemurafenib against BRAF and bortezomib against the proteasome.

Targeted therapy is a thriving area of cancer research, which is rapidly developing. As more novel targets will be identified by tumor DNA sequencing and other techniques, as more novel drugs may be developed to inhibit them. The scientific and economic impact of the concept of precision medicine will revolutionize cancer therapy in the years to come. Nevertheless, targeted drugs also have considerable disadvantages:

(1) Tumors frequently develop resistance against targeted drugs. Alterations in the target structure (*e.g*. point mutations), single nucleotide polymorphisms, cell cycle arrest, use of alternative signalling pathways or antigen shedding may cause ineffectiveness of treatment [9, 13].

(2) Unexpectedly, targeted drugs also reveal side effects in normal organs. Although the targets that are addressed by these drugs are not present in normal cells, there are nevertheless other non-specific off-target effects. Known side effects of targeted therapies include hepatotoxicity, dermatotoxicity (skin rash, hair depigmentation, nail changes), hypertension *etc*.

Natural products from plants and other natural origin provide an excellent resource for targeted therapies [45–51].

(1) The number of targets for therapeutic intervention is still increasing and novel drugs for novel targets need to be developed. Natural products may serve as lead compounds that can be chemically modified to generate derivatives with improved pharmacological properties.

(2) Natural products that overcome drug resistance. An example is the multidrug resistance (MDR) phenotype. Drug efflux pumps of the ATP-binding cassette (ABC) transporter family confer resistance to a broad spectrum of anticancer drugs including man targeted small molecules [52]. The lignin sesamin inhibits the ATPase activity of several ABC-transporters and is thus potentially more effective in overcoming MDR than previous MDR inhibitors that block only single efflux pumps [53].

(3) The severe side effects of classical cytotoxic (non-targeted) as well as of targeted therapy may be alleviated or abolished by natural products and medicinal plants (*e.g*. PHY906) [54–57].

**Figure 2:**
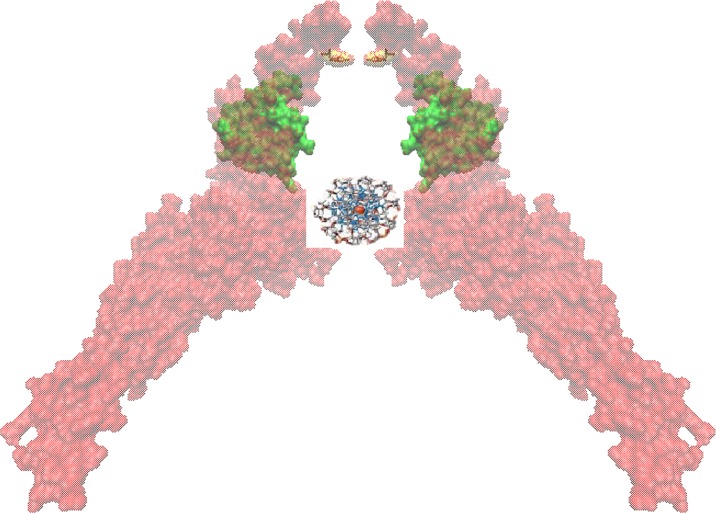
Homodimers of Stat3 bound to DNA for transcriptional activation SH2 domains are shown in green and the phosphorylation sites (Tyr705) are shown in yellow representation.

To illustrate the potential of natural products for targeted therapy, we have chosen STAT3. An important molecule in signal transduction processes of tumors represents signal transducer and activator of transcription 3 (STAT3). Upon binding of specific ligands (interferons, epidermal growth factor, interleukin-5 and -6) to their receptors, receptor-associated Janus kinases (JAK) are activated, which in turn activate STAT3. After phosphorylation of STAT3 at tyrosine at position 705, STAT3 dimerizes and translocates to the nucleus, where it binds to the DNA (Figure 2). As transcription factor, STAT3 induces the expression of genes involved in cell growth, apoptosis, invasion and metastatis, angiogenesis and other cellular processes. STAT3 activation can also take place by mitogen-activated protein kinases (MAPK) and c-SRC non-receptor tyrosine kinase by phosphorylation of the serine residue 727 of STAT3.

Constitutively activated STAT3 promotes carcinogenesis and STAT3 overexpression has been reported in many tumor types [58, 59]. Recent reports indicate that STAT3 may also act as tumor suppressor depending on the mutational background [60]. There are many efforts to disrupt the STAT3 signaling route by small molecule inhibitors [61]. Three major strategies have been used to reach this goal:

(1) Inhibitors acting upstream of STAT3: Tyrosine-kinase inhibitors of cell surface receptors (EGFR, HER2, PDGFR, IGFR, *etc*.) inhibit downstream signalling including the STAT3 pathway. The same is true for JAK 1/2, which are upstream of STAT3.

(2) STAT3 inhibitors that disrupt dimerization at the SH2 domain. The SH2 domain of STAT3 consists of the tyrosine-phosphorylation site at Tyr705 and two closely neighbored binding sub-pockets. Most STAT3 inhibitors bind to the SH_2_ domain [62].

(3) Inhibitors of the STAT3 DNA binding domain. The sterical hindrance by small molecules to bind to DNA inhibits the transcription factor activity of STAT3.

**Figure 3:**
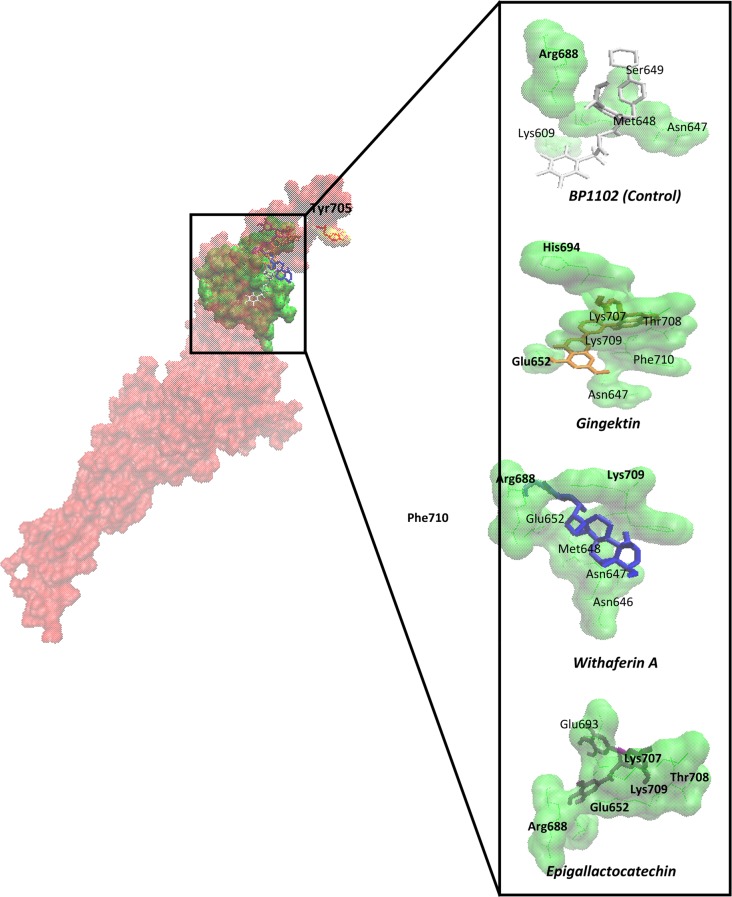
Defined molecular docking of phytochemicals to STAT3 at the SH2 domain STAT3 has been represented in surface format with red and SH2 in green. Phosphorylation site was shown in yellow (Tyr705). The compounds were shown in dynamic bond format with different colors. The binding residues were visualized closely. The residues that bound to compounds by hydrogen bond were shown in bold.

In addition to synthetic small molecules that have been described as STAT3 inhibitors, a number of phytochemicals from diverse medicinal plants also have been reported to block STAT3 phosphorylation and nuclear translocation (Table I). Molecular docking analyses demonstrate that they bind to the protein in a comparable manner as reported for synthetic small molecules (Table II). For comparison, the known STAT3 inhibitor BP-1-102 was used as synthetic control compound. Its binding energy was -7.46 (±0.36) kcal/ mol and the pKi value was 3.81 (±1.94) μM (Table II). Except of four natural products, all others revealed binding energies of lower than -6 kcal/mol. Six phytochemicals revealed binding energies even better than that of BP-1-102. The positions of the amino acids, which are involved in binding of the compounds to STAT3 indicate that they are located in the SH_2_ domain of the protein (Figure 3).

**Table I:** Phytochemical inhibitors of STAT3 tyrosine phosphorylation

Phytochemical	Plant	Reference
Curcumin	*Curcuma longa L.*	Bharti et al., 2003 [120]
Curcumin	*Curcuma longa L.*	Chakravarti et al., 2006 [121]
Cryptotanshinone	*Salvia miltiorhiza* Bunge	Shin et al., 2009 [122]
Cryptotanshinone	*Salvia miltiorhiza* Bunge	Ge et al., 2015 [123]
Epigallocatechin-3-gallate	*Camelia sinensis* (L.) Kuntze	Masuda et al., 2001 [124]
Epigallocatechin-3-gallate	*Camelia sinensis* (L.) Kuntze	Tang et al., 2012 [125]
(−)epigallo-catechin gallate	*Camelia sinensis* (L.) Kuntze	Senggunprai et al., 2014 [126]
Honokiol	*Magnolia officinalis* Rehder & Wilson	Rajendran et al., 2012 [127]
Honokiol	*Magnolia officinalis* Rehder & Wilson	Saeed et al., 2014 [128]
Resveratrol	various species	Bhardwaj et al., 2007 [129]
Resveratrol	various species	Yu et al., 2008 [130]
Cucurbitacin B	*Cucumis melo* L.	Yang et al., 2016 [131]
Cucurbitacin I	*Curcurbita andreana*	Blaskovich et al., 2003 [132]
1,2,3,4,6-penta-O-galloyl-beta-D-glucose	*Paeonia suffruticosa* Andrews	Lee et al., 2011 [133]
Withacnistin	*Acnistus arborescens* Schltdl.	Zhang et al., 2014 [134]
Piperlongumine	*Piper longum* L.	Bharadwaj et al., 2015 [135]
Guggulsterone	*Commiphora mukul*, (Arn.) Bhandari	Leeman-Neill et al., 2009 [136]
Matrine	*Sophora flavescens*, Aiton	Yang et al., 2015 [137]
Eriocalyxin B	*Isodon eriocalyx* (Dunn) Kudo	Yu et al., 2015 [138]
Ginkgetin	*Ginkgo biloba* L.	Jeon et al., 2015 [139]
Angoline	*Zanthoxylum nitidum* (Roxb.)DC	Liu et al., 2014 [140]
Withaferin A	*Withania somnifera* (L.) Dunal	Yco et al., 2014 [141]
Chrysin	Propolis (bee glue)	Lirdprapa-Monkol et al., 2013 [142]
Icariside II	*Epimedium koreanum* Nakai	Kang et al., 2012 [143]
Licochalcone A	*Glycyrrhiza inflata* Batalin	Funakoshi-Tago et al., 2008 [144]
Quercetin	various species	Senggunprai et al., 2014 [126]
BP-1-102	synthetic control compound	Zhang et al., 2012 [145]

**Table II:** Defined molecular docking of natural products to the SH2 domain of STAT3

Compound	Binding Energy (kcal/mol)	pKi (μM)
Gingektin	−9.16 ± 0.20	0.20 ± 0.06
Withaferin A	−8.89 ± 0.15	0.31 ± 0.07
Epigallocatechin-3-gallate	−8.49 ± 0.29	0.65 ± 0.27
Cucurbitacin B	−7.87 ± 0.56	0.39 ± 0.16
Cucurbitacin I	−7.81 ± 0.11	1.90 ± 0.38
Withacnistin	−7.54 ± 0.09	3.00 ± 0.46
BP-1-102**	−7.46 ± 0.36	3.81 ± 1.94
Guggulsterone	−7.29 ± < 0.001	4.51 ± 0.01
Licochalcone A	−7.02 ± 0.06	7.12 ± 0.65
Angoline	−7.01 ± 0.02	7.25 ± 0.24
Curcumin	−7.00 ± 0.14	7.49 ± 1.63
Piperlongumine	−6.98 ± 0.09	7.76 ± 1.24
Neoambrosin	−6.96 ± 0.01	7.95 ± 0.12
Damsin	−6.83 ± < 0.001	9.86 ± 0.01
Eriocalyxin B	−6.82 ± 0.01	9.97 ± 0.25
Quercetin	−6.63 ± 0.04	13.69 ± 0.88
Chrysin	−6.60 ± 0.03	13.90 ± 1.09
Resveratrol	−6.23 ± 0.16	27.76 ± 7.96
Icariside II	−5.91 ± 0.65	33.07 ± 16.46
Matrine	−5.86 ± < 0.001	50.58 ± 0.01
Pentagalloylglucose	−2.60 ± 0.16	12,625 ± 3,217.34
Cryptotanshinone	−2.47 ± 8.56	3.69 ± 0.01

Lowest binding energies and predicted inhibition constants (pKi) have been shown. Each docking experiment has been repeated three times.

** Synthetic drug used as control compound for comparison [131].

Natural products and many synthetic drugs as well are rarely mono-specific and frequently address more than one target [63]. In addition to binding to one target protein, downstream signaling is affected so that it can be assumed that rather many than single proteins or genes are involved in the modes of action of a drug. Deciphering the full complexity of cellular mechanisms and signalling pathways affected by a drug can be a tedious task.

## NETWORK PHARMACOLOGY WITH NATURAL PRODUCTS, MEDICINAL PLANTS AND COMPLEX HERBAL REMEDIES

**Figure 4:**
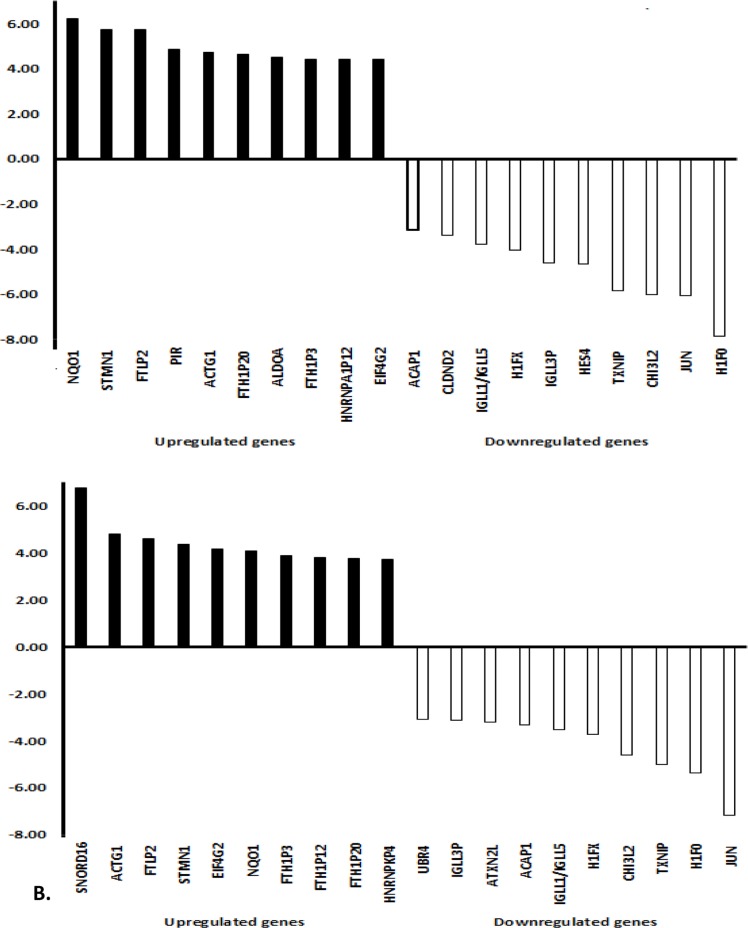
Top 10 increased and top 10 decreased genes in CCRF-CEM leukemia cells treated with neoambrosin or damsin from *Ambrosia maritima* Leukemia cells CCRF-CEM were treated with both compounds for 24 h. Afterwards the mRNAs were extracted and subjected to microarray hybridization on Illumina Human HT-12 Bead Chip arrays after cDNA synthesis and labelling. Microarray scanning was done using an Illumina^®^ Bead Station array scanner (Illumina, San Diego, CA, USA) at Genomics and Proteomics Core Facility at the German Cancer Research Center (DKFZ, Heidelberg, Germany). The data were analyzed using Chipster software, subsequent assessment of significant genes was performed using empirical Bayes t-test (p < 0.05) with Bonferroni correction. Statically significant genes were further analyzed into Ingenuity Pathway Analysis software (IPA; Ingenuity Systems, Redwood City, CA, USA) to determine cellular networks and functions affected by each compound.

**Figure 5:**
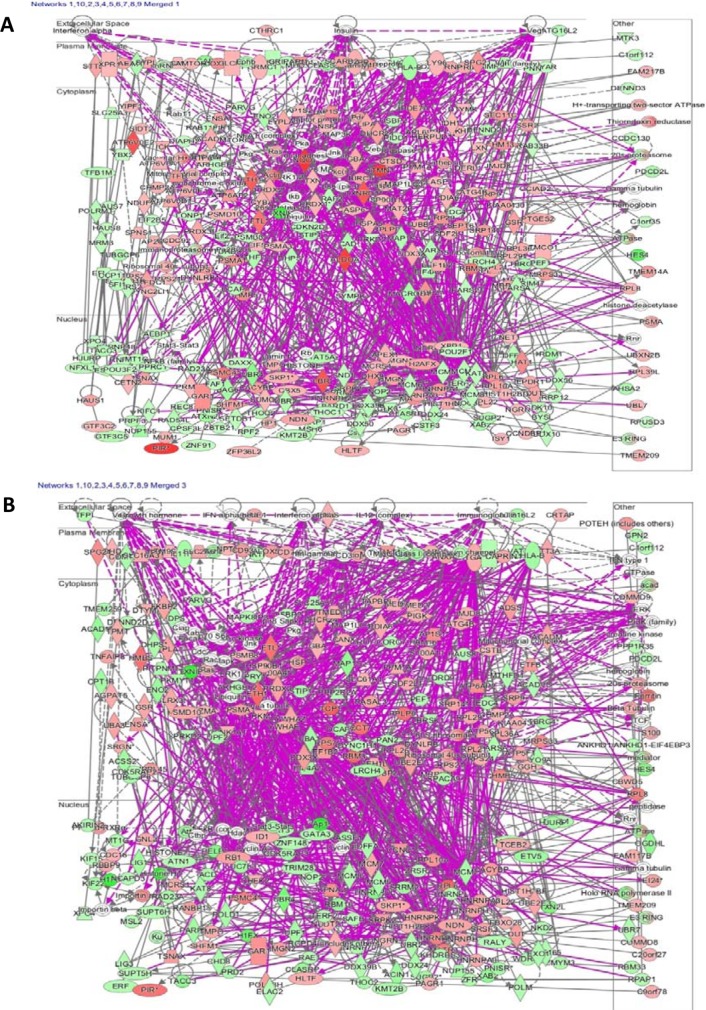
Top 10 out of 25 networks of CCRF-CEM leukemia cells affected by treatment with (A) neoambrosin or (B) damsin from *Ambrosia maritima* Cellular molecules are represented as nodes and the biological relationship between two nodes is represented as a line. The intensity of the node color indicates the degree of up-(red) or down-(green) regulation. Solid lines show direct, dotted lines and indirect actions. Gray lines show actions within one network, purple lines show actions between different networks. The networks of neoambrosin and damsin involved RNA post-transcriptional modification, cellular assembly and organization, cellular function and maintenance, cell cycle, molecular transport, RNA trafficking, DNA replication recombination and repair, cellular growth and proliferation, as well as cell death and survival. In addition, the affected pathways for neoambrosin were NRF2-mediated oxidative stress response, EIF2 signaling, cell cycle control of chromosomal replication and protein ubiquitination pathway, whereas for damsin NRF2-mediated oxidative stress response, EIF2 signaling ephrin receptor signaling, role of JAK2 in hormone-like cytokine signaling, JAK/Stat signaling were the top affected pathways.

As an example to illustrate the complexity of pharmacogenomics networks, we have chosen neoambrosin and damsin, two cytotoxic phytochemicals from the medicinal plant, *Ambrosia maritima* L. Their cytotoxic activity towards cancer cells has been reported [64]. In addition to targeting STAT3 (Table II, Figure 3, [64]), microarray analyses revealed that the expression of numerous genes was increased or decreased by these two compounds. The assignment of these genes to their corresponding signaling pathways unraveled many different actions, which all may contribute to different extent to the bioactivity of these compounds. The network analyses in Figures 4 and 5 illustrate the complex interaction networks of these two compounds. Microarray analyses revealed that the mRNA expression of 606 and 473 genes was increased or decreased after treatment of CCRF-CEM leukemia cells with neoambrosin and damsin, respectively. Figure 4 shows each the top 10 most increased or decreased genes upon treatment of cells with neoambrosin or damsin. STAT3 was also among the increased genes. These genes have been subjected to Ingenuity network analysis (Figure 5). One of the advantages of network pharmacology is that all possible actions can be identified in a comprising manner. However, at the same time this bears the danger that genes appear, which are not causatively related to the mechanism of action of a drug. The art is to separate non-relevant background noise from those signals that really contribute to the modes of action by subsequent experimentation. Genetic networks such as those shown in Figure 5 rather represent the start than the end of processes to elucidate the mode of action of drugs.

As a consequence of the complexity of drug actions, another new concept is emerging called polypharmacology, which focuses on drugs attacking multiple instead of single targets to perturb disease-associated networks [65, 66]. To combat complex systemic diseases such as cancer, single target drugs have been proven to be less effective than promiscuous compounds that influence multiple targets and exert maximal efficacy and minimal toxicity [67–70]. The analysis of complex signaling network may unravel novel targets for drug development [71, 72]. Network-based approaches are rapidly emerging in recent years [65, 68, 73]. Polypharmacology might also provide novel opportunities to fight drug-resistant tumor cells [74]. Network pharmacology facilitates the establishment of pragmatic network models and the prediction of drug targets based on public databases. In addition, it also helps to construct predictive ‘*drug target disease*’ network models using high-throughput screening and bioinformatics. Such approaches help to investigate underlying mechanisms of drug actions on biological networks by comparing the action of a drug with its target [75].

Cancer is a complex disease arising from changes in multiple biological networks [76], which is believed to require complex therapeutic approaches [77]. Finding drugs that act in multiple pathways, or to discern possible drug combinations represents one of the main future challenges by understanding the signaling networks of human cells and how they are altered in different cancers [78].

To be highly effective, interventions within biological networks should be multiple on the one hand, but extremely selective on the other hand to spare normal organs from detrimental side effects [79]. Holistic approaches may qualify network pharmacology as suitable tool for drug development for complex diseases such as cancer.

There are various sophisticated signaling networks driving tumorigenesis and tumor progression that can be therapeutically targeted. Instead of initial time-consuming laboratory experiments, network biology approaches for well-known pathways with various drugs might be more suitable for timely cancer drug discovery [80].

Network pharmacology facilitates the identification of new signaling networks that are distorted in various cancer types. Recently, Jaeger et al. discovered several known but unexpected as well as unknown pathways in triple negative breast cancer [81] and in ovarian carcinoma [82]. Pan-cancer network analysis of mutated networks helps to identify mutated sub-networks in cancer and paves the way to investigate new diagnostic and therapeutic prospects for specific cancer subtypes [83].

In the past few years, numerous novel signaling networks have been unraveled that contribute to carcinogenesis and tumor progression. It becomes more and more clear that the complex nature of alterations in cancer cells can hardly be attacked by drugs addressing single targets or pathways. Comprising, novel therapeutic strategies have to be devised that are able to inhibit entire malignancy-regulating networks. It is hoped that network pharmacology provides a platform for the development of a novel generation of drugs fulfilling this requirement.

Network pharmacology became a powerful tool to systematically reveal complex biological relationships. In cancer and other diseases, network pharmacology relies on “-omics” approaches to detect the variables at fundamental cellular and molecular units in response to the specific pathophysiology and/or drug treatment. The generated dataset of variables helps to generate networks from the genomic up to the metabolomic level to classify molecular processes in disease conditions. The high availability of multi-omics cancer databases opens new opportunities for data integration that promises an in-depth understanding of cancer and clinically and biologically meaningful tumor stratification [84].

The “-omics” technologies allow to measure not only well-known signaling pathways and biological networks in its entirety, but also to detect novel pathways and mechanisms that have not been described before in a given experimental context. Another considerable advantage is that cellular alterations caused by even highly complex herbal mixtures can be measured. Frequently, the holistic approach of herbal medicine cannot be satisfactorily investigated by the reductionistic methods of western science. Novel methods of network pharmacology may offer new solutions for holistic approaches.

On the other hand, some disadvantages have to be considered. With huge amounts of generated data, it may be difficult to distinguish causative mechanisms from irrelevant “background noise”. Additional verification experiments are indispensable to substantiate the results obtained from “-omics” technologies. In the past, time and concentration kinetic experiments have rarely been performed in network pharmacology, because of the high costs. This bears the danger that optimal time points and drug concentrations to measure a pharmacological effect may be missed. With decreasing prices and further technology developments, this disadvantage might be overcome. Another general disadvantage is that expression analyses (*e.g*. transcriptomics or proteomics) do not allow to draw conclusions about the functional state of proteins (*e.g*. their phosphorylation state). This emphasizes the necessity to validate pathways identified by expression analyses by additional experiments.

Recently, there is a shift from microarray technologies to next generation sequencing methods such as RNA sequencing. In contrast to microarrays, this technology is not limited by the existing of sequencing information of genes. Another advantage is that background signals are much lower in RNA sequencing compared to microarray hybridization. Furthermore, the dynamic range for quantification of gene expression is much better leading to high levels of reproducibility among both technical and biological replicates.

Approaches with pleiotropic natural products that target multiple proteins and pathways in cancer-associated networks may be promising. Traditional herbal medicines play an important role in health maintenance all over the world. Herbal medicines are regarded as a valuable resource for new active compounds in drug discovery due to their multiplicity in structure, bioactivity and tolerability. The concept of ‘*one disease - one drug - one target*’ is shifting towards ‘*one disease - one drug - multiple target*’ [65, 85–88]. Network-based strategies will facilitate structure-based drug design, forecast harmful side effects of drugs, and predict the effects of drug-binding on biomolecules and signaling pathways [89].

Tumors frequently develop resistance to mono-specific drugs. Mutations in the corresponding target protein may easily lead to inefficacy of such a drug. By contrast, drugs addressing multiple targets are not compromised in their activity, if mutations in one of the targets appear. Most natural products exert their bioactivity by attacking multiple rather than single targets [63]. It can be speculated that during evolution of life the selective pressure favoured the emergence of multi-target specific compounds, as they make organisms more successful and competitive in the struggle for life.

Network pharmacology may provide unique opportunities for systematic target identification and possibilities to address them by multi-target specific natural compounds [90]. Highly connected nodes in complex protein networks are more vulnerable for pharmacological inhibition of the entire network than other nodes [91]. However, not all protein nodes in a network can be inhibited by drugs. It has been estimated that only about 15% of any protein nodes in a given network are druggable. To generate rational phytotherapies based on network information, several strategies can be considered:

(1) Plants or herbal mixtures can be considered, if their bioactive chemical constituents are known. This approach is based on their use in traditional medicines and is largely experience-based. In a way, herbal mixtures are comparable to multidrug combination therapy with synthetic drugs and polypharmacology [92].

(2) Multi-target specific therapy may also be reached with single phytochemicals with selective polypharmacological approaches [93, 94]. The promiscuity of many drugs to react with more than one target has been negatively discussed in the past as off-target activities. In the context of network pharmacology, off-target effects may be reinterpreted as broader polypharmacology profiles.

(3) Recent concepts in network pharmacology emphasize the potential of synthetic lethality [95]. Proteins which a non-essential in normal cells may reach therapeutic relevance, if connected in a cancer network [96]. Their combined elimination or inhibition may lead to improved or even synergistic tumor cell eradication. Many single gene or protein knockouts exhibit no or marginal effects on tumor growth, even if relevant cancer-related targets are affected. In normal tissues, redundant protein functions and compensatory signal transduction pathways lead to robust phenotypes [97]. What makes much sense in normal physiology of organisms, poses severe treatment obstacles in cancer therapy. A conceptual solution to this problem may be not to knock out single disease-causing proteins, but to perturb entire disease-causing networks by polypharmacology with phytochemicals or complex herbal mixtures targeting multiple targets in cancer networks.

A network pharmacology approach for green tea polyphenolics revealed their multiple bioactivities towards various diseases including cancer. A total of 200 human targets were identified. This study illustrated the mechanisms of pleiotropic activity of green tea polyphenolics towards cancer, diabetes, neurodegenerative disease, cardiovascular disease, muscular disease, and inflammation [98]. Recently, a novel integrated Herbal Medicine Systems Pharmacology (HMSP) platform was used to investigate how herbs act on the human body at the molecular level. This platform supports drug absorption, distribution, metabolization and excretion (ADME) prediction, target fishing, drug target generation and data processing for the association of herbs’ actions to diseases and organisms [99]. Using another systems pharmacology method, the mechanism of restoration of proper balance and harmony inside the body, organ and energy system has been elucidated for *Qi*-enriching and blood-tonifying herbs in TCM [100].

Network pharmacology may also help to better understand the reasons for failure of drugs in clinical trials regarding clinical efficacy, side effects and toxicity. The world populations are heterogeneous and genetic polymorphisms in pharmacologically relevant genes varying across geographical region are significant [101]. In addition, tumors are also heterogeneous, which is defined by presence or absence of actionable therapeutic targets [102]. The currently available therapeutic interventions for heterogeneous populations with heterogeneous tumors are still very limited. Network pharmacology approaches may overcome the pitfalls in cancer therapy and facilitate the development of novel anticancer drugs.

## CONCLUSIONS AND PERSPECTIVES

The fact that chemosensitivity testing was not established as routine laboratory method may have historical reasons. In times as combination therapy protocols were established as being superior to monotherapy, data showing that tumors can reveal resistance towards many drugs at the same time was compromising the utility of drug combination regimens.

On the one hand, clinical experiences showed combination therapy protocols were able to improve treatment results, but sustainable use of patients from cancer is far from reality in many cases. On the other hand, molecular mechanisms were discovered in basic sciences that explained the appearance of broad spectrum resistance phenomena (*e.g*. ABC-transporter-mediated multidrug resistance, apoptosis resistance *etc*.). A re-thinking may lead to a revival of predictive testing. Rather than prediction of drug sensitivity (which is not sufficiently reliable), drug resistance can be predicted with high precision. With multi-modal treatment options at hand, the knowledge about high probability that a specific drug would fail in a specific patient is valuable, because it allows early to switch to other more effective drugs or therapy strategies.

The development of resistance and the severe side effects of classical cytotoxic cancer therapy lead to a paradigm shift from the poorly specific cytotoxic anticancer drugs to targeted drugs, which were expected to be more tumor-specific. Indeed, this turned out to be a thriving concept with numerous new drugs on the market, which of course did not replace the classical cytotoxic drugs, but did supplement the armory to fight cancer. Although treatment outcomes could be further improved by targeted drugs they unfortunately also reveal side effects and are subject to resistance development. In this context, the therapeutic potential of phytochemicals cannot be overseen. They were already valuable in the era of cytotoxic drugs. Paclitaxel, vincristine, camptothecin, etoposide are just a few examples for established plant-drived anticancer drugs. There is a plethora of literature demonstrating that phytochemicals are also valuable for targeted cancer therapy.

Cancer stem-like cells are rare self-renewing omnipotent cells, which proliferate upon appropriate stimulation and differentiate into heterogeneous lineages in tumors. They are frequently resistant to conventional chemo- and radiotherapy. Interestingly, natural products have been described to inhibit cancer stem-like cells [103–107]. This is a new field of research that is worth being investigated in more detail to understand the full range of mechanisms, which are responsible why some natural products are able to attack stem-like cells.

With the recent developments in bioinformatics, network pharmacology emerges as novel concept in therapy research. Phytotherapy with mixtures of several herbs as well as isolated single compounds exert their bioactivity by targeting multiple sites in diseased cells. A challenge for research in network pharmacology will certainly be to extract meaningful information from thousands of data points. What is a mechanistically relevant signal and what is background noise? Finding the needle in the haystack will be a task for the future and smart computer algorithms are required. Network pharmacology has to cope with multiple dimensions of problems. The multi-targeted nature of drug action, resistance development, side effects on normal organs and tissues, inter-individual biological variations, as well as inter- and intra-tumoral differences have to be considered. Especially the problem of tumor heterogeneity and possibilities to tackle with genetically diverse tumor subpopulations deserve attention from our point of view. Heterogeneous tumor populations represent a main reason for the development of resistant refractory tumors. Resistance also prevents to apply doses high enough to kill all cells, because of the severe side effects of anticancer drugs. Therefore, novel strategies to eradicate heterogeneous tumor subpopulations might not only fight the development of drug resistance but also facilitate to reduce side effects.

**Figure 6:**
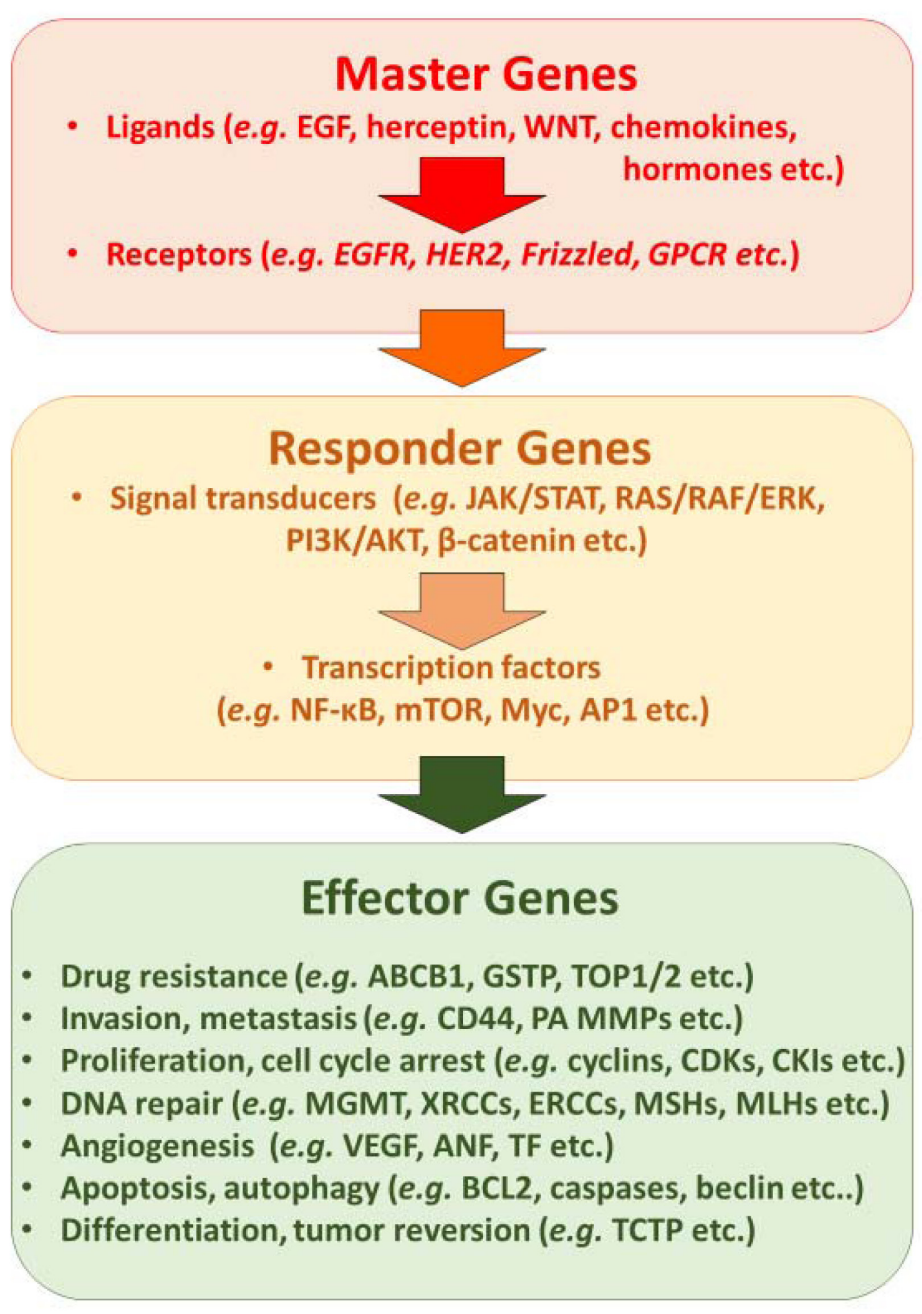
Synopsis of main molecular mechanisms that can be targeted by synthetic small molecules, therapeutic antibodies and natural products

Integrating precision medicine into routine cancer therapy is certainly one of the predominant tasks of the next years to come (Figure 6). To realize this concept, it is not only necessary to establish the scientific basis allowing routine application in the clinic, but also to develop and integrate economic models, which allow the implementation of personalized medicine [108–111]. This is true independent of whether synthetic drugs or phytotherapeutic approaches will be used. While there is a plethora of literature on the preclinical activity of phytochemicals and medicinal plant preparations, results from clinical trials are still relatively rare. However, there are well-done clinical trials that provide evidence that phytochemicals and plant preparations are indeed active in the clinical setting [111–119]. For the sake of future patients, health care systems in industrialized and developing countries should do any effort to improve cure rates of tumor diseases.
